# Patchy promiscuity: machine learning applied to predict the host specificity of *Salmonella enterica* and *Escherichia coli*

**DOI:** 10.1099/mgen.0.000135

**Published:** 2017-10-03

**Authors:** Nadejda Lupolova, Tim J. Dallman, Nicola J. Holden, David L. Gally

**Affiliations:** ^1^​ University of Edinburgh, Edinburgh, UK; ^2^​ Public Health England, England, UK; ^3^​ James Hutton Institute, Dundee, UK; ^4^​ Division of Immunity and Infection, The Roslin Institute, University of Edinburgh, Easter Bush, Edinburgh EH25 9RG, UK

**Keywords:** host specificity, machine learning, Support Vector Machine, *Salmonella*, *E. coli*, zoonosis

## Abstract

*Salmonella enterica* and *Escherichia coli* are bacterial species that colonize different animal hosts with sub-types that can cause life-threatening infections in humans. Source attribution of zoonoses is an important goal for infection control as is identification of isolates in reservoir hosts that represent a threat to human health. In this study, host specificity and zoonotic potential were predicted using machine learning in which Support Vector Machine (SVM) classifiers were built based on predicted proteins from whole genome sequences. Analysis of over 1000 *S.* *enterica* genomes allowed the correct prediction (67 –90 % accuracy) of the source host for *S*. Typhimurium isolates and the same classifier could then differentiate the source host for alternative serovars such as *S*. Dublin. A key finding from both phylogeny and SVM methods was that the majority of isolates were assigned to host-specific sub-clusters and had high host-specific SVM scores. Moreover, only a minor subset of isolates had high probability scores for multiple hosts, indicating generalists with genetic content that may facilitate transition between hosts. The same approach correctly identified human versus bovine *E. coli* isolates (83 % accuracy) and the potential of the classifier to predict a zoonotic threat was demonstrated using *E. coli* O157. This research indicates marked host restriction for both *S. enterica* and *E. coli*, with only limited isolate subsets exhibiting host promiscuity by gene content. Machine learning can be successfully applied to interrogate source attribution of bacterial isolates and has the capacity to predict zoonotic potential.

## Abbreviations

MLST, multilocus sequence typing; PV, protein variant; ΔPV30, subtractive difference equal to 30 or less in proportions of a PV between two classes; ST, sequence type; STm, Salmonella Typhimurium; stx, Shiga toxin alleles; SVM, Support Vector Machine.

## Data Summary

1. Data used for this work can be downloaded from https://figshare.com/s/7a3ededa8cedd95b9fb7. The files include isolate IDs, protein variants (PVs) and their annotations for *Salmonella enterica* and *Escherichia coli*.

2. Descriptive PVs for each model also can be found at https://figshare.com/s/7a3ededa8cedd95b9fb7. The name of the file describes the model for which these PVs were used. So salmonella_PV_30_AO_annotations.csv means these are the PVs that describe *Salmonella* Typhimurium Avian isolates vs all Other isolates.

3. Isolate metadata for both species (original host, predictions, place, year and multilocus sequence type) are visualized using pan genome trees and can be viewed on ITOL: http://itol.embl.de/shared/nlupolova.

## Impact Statement

Both *Salmonella enterica* and *Escherichia coli* are bacterial species with a broad animal host range with strains that can colonize humans and in some cases cause lethal infections. Both species have large accessory genomes and it is established for certain subtypes (serovars) of *S. enterica* that these can be host-restricted with both gene acquisition and loss contributing to the degree of host specificity. The extent of host restriction for *E. coli* and *Salmonella* serovar Typhimurium is not known and the capacity to predict the source of human infections with these bacteria is important to understand the origin of zoonoses and aid public health interventions. The work in this study has successfully applied a machine learning algorithm, Support Vector Machine, to attribute the source animal or environment of these bacteria based on their genome content. The work should have value to allow the sources of zoonotic outbreaks to be identified and also to assign sources to environmental and water pollution events. The research will also help identify the genes and pathways that lead to host restriction and are therefore required for infection in different animal hosts.

## Introduction

*Salmonella enterica* and *Escherichia coli* can be isolated from a large number of animal hosts, in particular birds and mammals. When isolated, *S. enterica* serovars are usually associated with disease whereas the majority of *E. coli* are commensals with only a subset considered overt pathogens [1, 2]. Infections caused by these two genera are a major burden on human morbidity and mortality and many of these infections are zoonotic, i.e. are transmitted from animals to humans. Host restriction or specificity has been a key area of research for *Salmonella*, and host-specific serovars such as *S*. Typhi and *S*. Gallinarum are responsible for more severe systemic disease in their primary host, whereas serovars with broader host ranges, such as *S*. Typhimurium (STm) and *S.* Enteritidis are often restricted to gastrointestinal disease in their different hosts. However, this differentiation is increasingly appearing simplistic with identification of invasive strains of STm, such as ST313, in humans [3–5]. The fundamental biology underlying host restriction is important to understand as it shows the barriers these bacteria need to overcome to successfully colonize and cause disease in a new host. From a public health perspective, the capacity to ascribe correctly the source of an infection is important as it can inform ways to intervene and limit human infection from animal and food sources.

Compared to *S. enterica*, the host-specificity of *E. coli* has been less well investigated. Classically the species has been divided into phylogroups (A, B1, B2, C, D, E and F) which are based on possession of a small number of specific alleles [6]. This classification was considered to have only a weak association with isolation host but has the advantage that commensal and pathogenic isolates are often assigned to separate types [7]. The genetic relatedness demonstrated by a reduced allele methods such as multilocus sequence typing (MLST) and phylogroup agree well with high-resolution core genome SNP typing [8, 9]. More recent studies that also take into account accessory genome information do provide examples of host specialization for *E. coli* [10, 11], which should also include *Shigella* species as a type of enteroinvasive *E. coli* [2, 12]. Fundamentally, *Escherichia* and *Salmonella* share the same gene acquisition, mutation and recombination systems as well as overall physiology. As such both should have the same genetic potential for plasticity that could result in host adaptation or host promiscuity. In the last few years short read sequences from thousands of bacterial genomes have been deposited in databases, although often their use is limited by lack of associated metadata. Where the isolation host is known, this now provides an opportunity to interrogate such sequence data for genetic signals and predictors of host specificity and determine how these map onto the phylogeny of the different species. Recently, a machine-learning algorithm, Support Vector Machine (SVM), was used to analyse sequence data from *E. coli* O157 isolated from cattle and humans, so as to determine if all cattle isolates had the same genetic potential to cause detectable infections in humans [13]. There were isolates from cattle that had genetic information allied more closely to isolates associated with human infection, indicative of strains with increased zoonotic potential. In the current study, we have combined a machine learning approach with pan genome analyses of both *S. enterica* and *E. coli* to investigate the relatedness of isolates from different hosts. The primary aim of this study was to demonstrate the potential of machine learning, in this case SVM, to predict the source of an isolate, and indicate its potential to transfer between hosts, including the zoonotic threat to humans.

## Methods

### Genome analysis

Illumina short read sequences were assembled with SPAdes [14] and annotated with Prokka [15]. Sequence type was assigned using MLST v. 2.4 [16]. Pan genomes were clustered with Roary [17], paralogues were split and the threshold for sequence similarity was set to 95 % at the amino-acid level. The core SNP trees were built with RAxML [18] based on aligned core genes (Table 1). Accessory trees based on the presence or absence of accessory genes were extracted from the Roary output (Table 1). Shiga-toxin (*stx*)-positive isolates were detected using a blastn search with an *stx1* query (NC_004913.3, coordintates: 33251–31917) and *stx2a* query (NC_002695.1, coordinates: 1266960–1267928).

### Support vector machine analysis

SVM implementation in R package e1071 [19] was used to build classifiers with radial kernel, weighted classes, and ‘gamma’ and ‘cost’ parameters adjusted after tuning for each host. Protein presence and absence output from Roary were used to identify features for each class of an SVM model. Proteins were clustered with high (95 %) similarity, and therefore related proteins (less than 95% similarity) were allocated into different clusters. For these the term ‘protein variants (PVs)’ was introduced to more precisely describe the Roary output. PVs that differentiate the two classes under test were chosen for the respective classifier. For example, the proportion of each PV found in the STm avian host group was compared with the proportion found in the ‘all others’ STm host groups. PVs which differed by at least 30 % between the two groups (ΔPV30) were used as descriptive features for the SVM STm model. The higher the ΔPV values the more clearly distinct the groups are, although there is a trade-off between PV discrimination, the number of PVs and model accuracy (Figs S1 and S2, available in the online Supplementary Material).

All SVM classifiers in this study were based on comparing two groups of data. For example, for serovar Typhi vs Dublin this was straightforward and the two training classes were based on the predicted proteins extracted from these two specific serovars. For analysis of STm and for *E. coli* datasets a ‘one against all’ approach [20] was used. For each classifier, the differential PVs are defined by comparing PVs of the isolates from one specific host with those from isolates from the remaining hosts combined. This means that for STm four different classifiers were built (avian vs the rest; cattle vs the rest; human vs the rest; and porcine vs the rest) and for *E. coli* six different classifiers were used (human vs the rest; porcine vs the rest; canine vs the rest; avian vs the rest; environmental vs the rest; and cattle vs the rest).

For each classifier 10× cross-validation was performed, meaning the data were split randomly into 10 groups and trained on 90 % of the isolates and the remaining 10 % used for testing, and this process was then repeated with different gamma and cost values. Different approaches to sub-sampling for test sets were taken. The main method was to remove each isolate in turn from the training set; each time PVs were re-calculated, the model was tuned and parameters were adjusted. After this the removed isolate was tested. From this a probability attribution for each isolate and for each host was generated. The overall performance for each classifier was assessed by plotting true positive vs true negative rates and calculating an area under the curve for each of the classifiers (Fig. S3 for STm and Fig. S4 for *E. coli*).

The Typhi and Dublin datasets were analysed differently. (1) Initially 20 isolates were randomly selected from each Typhi-human and Dublin-bovine dataset to be used as the test groups. The remaining Typhi-human and Dublin-bovine isolates (training dataset) were labelled and differential PVs were calculated between them. ΔPV90 values were used as features for the model. To access model accuracy 10× cross-validation was performed on the training set and the best parameters of ‘cost’ and ‘gamma’ were extracted from the tuning step. (2) A second test was similar to the above except that for the test group we included four Dublin human isolates. (3) To test the Dublin serovar alone, one isolate was removed for each assessment cycle, and the SVM classifier was retrained on all other human and bovine Dublin isolates; this involved recalculating discriminatory PVs (with ΔPV50 for the model) and then testing the removed isolate. (4) The combination involved the STm human and bovine datasets as a training model with ΔPV30, and then all isolates from the Dublin-bovine, Dublin-human and the Typhi datasets were tested.

Significance of the results was described using *P* values, which were obtained using basic R functions as required: Student's *t*-test (t.test function) and Fisher's exact test (fisher.test function).

## Results

###  *Salmonella enterica*

In total, 1682 *Salmonella* sequences were obtained from Enterobase [21]. The collection included serovar Typhi (250 human isolates), serovar Dublin (187 bovine isolates and 40 human isolates), and serovar Typhimurium (STm; 336 human isolates, 300 bovine isolates, 311 avian isolates, 256 swine isolates) (defined in supplementary file ‘Salmonella_data.tgz’). The isolates were diverse in terms of their year of isolation, ranging from 1945 to 2016, and their geography, which covered all continents with the exception of Antarctica. We assigned sequence type (ST) based on the *S. enterica* MLST scheme and identified 52 different STs in the whole dataset, although for each serovar one or two STs were dominant: Typhi ST 1 (*n*=185); Dublin ST 10 (*n*=206); and Typhimurium ST 19 (*n*=992) and ST 34 (*n*=122).

**Fig. 1.**
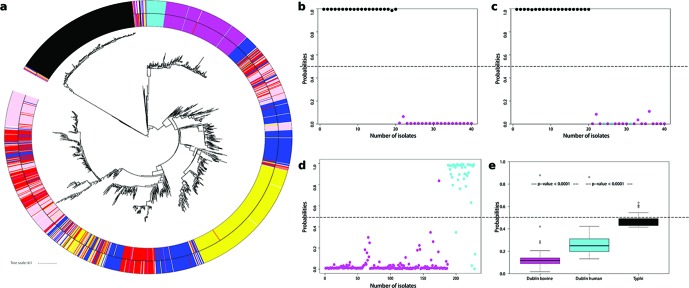
Host association of *Salmonella enterica*. Colour scheme of serovars: Typhi (black); Dublin-bovine (magenta); Dublin-human (cyan); STm avian (yellow); STm bovine (red); STm human (blue); STm swine (pink). (a) Clustering of isolates based on accessory genome content (non-core): distinct branches are evident for Typhi and Dublin serovars. Inside of STm there is some clustering associated with host; the majority of avian isolates cluster together, 80 % of the human isolates cluster in three groups, while the bovine and swine isolates are mostly found in groups of mixed origin. The outer ring shows the SVM host prediction when >0.5 (see Methods) and is otherwise left blank. (b) SVM prediction of *Salmonella* Typhi (human) vs serovar Dublin (bovine). Twenty isolates were randomly taken from each serovar for testing, and the model was trained on the remaining sequences (230 Typhi-human, 167 Dublin-bovine). Prediction was 100 % accurate due to highly discriminatory PVs (∆PV90=1349, ∆PV100=8). (c) The SVM classifier in (b) was applied to serovar Dublin isolates from both cattle (magenta) and humans (cyan): this primarily discriminates the serovar not the host as there is still complete separation between Typhi (black) and Dublin serovars (cyan and magenta). (d) If predictions were based on training with only Dublin human and bovine information then the Dublin isolates can be separated by this classification. (e) In this case STm bovine and human isolates were used as the training sets and testing was carried out on the distinct serovars: Dublin-bovine, Dublin-human and Typhi-human. Notably, the three groups can now be separated by the STm classifier in a logical trend based on isolation host.

Both core (3175 genes) and accessory (20 132 genes) genome relationships (Table 1) were plotted as trees based on information derived from the sequences. The clustering obtained for both trees (Figs 1 and S5) was quite similar with serovars Typhi, Dublin and STm on separate branches, illustrating a good correlation between core SNPs and accessory genome content at serovar level. Overall, both core and accessory trees show a large avian cluster that contained 82 % of all the avian STm isolates, a few different human clusters with the largest two in the accessory tree containing 38 and 20 % of all human STm isolates and one bovine cluster with 23 % of all bovine STm isolates (Fig. 1). Swine isolates had no sub-cluster that contained at least 20 % of the isolates together in the accessory tree but the core tree did contain such a cluster. The accessory genome therefore indicates some clustering by host for STm, especially for avian and human isolates, but many of the STm isolates from different hosts were interspersed within several branches containing isolates of mixed origin. *S*. Typhi and *S*. Dublin were included as ‘established’ host-restricted serovars and provided a framework for analysis of the host association of STm isolates.

**Table 1.** Summary of gene content for *S. enterica* and *E. coli* isolates analysed in this study

Section	Description	*S. enterica*	*E. coli*
Number of isolates		1682	943
Core genes	99 %≤strains≤100 %	3175	1328
Accessory genes	0 %≤strains≤99 %	20 132	91 087
Soft core genes	95 %≤strains<99 %	236	815
Shell genes	15 %≤strains<95 %	2098	3516
Cloud genes	0 %≤strains<15 %	17 748	86 746
Total genes	0 %≤strains≤100 %	23 307	92 415

#### SVM prediction of isolation host

To predict the isolation host of an *S. enterica* isolate, the SVM classifier was built using a ‘one against all’ approach, i.e. differentiating one host group from all other host sequences based on discriminatory PVs as described in the Methods. Initially, the classification and prediction method was applied to serovars Typhi and Dublin. *S*. Typhi is human host-specific while *S*. Dublin is generally associated with severe infections in cattle with some human cases. There were 752 ΔPV90 found almost exclusively in the Typhi isolates and similar numbers (*n*=746) describing the Dublin isolates. Randomly taking 20 isolates from each serovar for testing, and training on the remainder, it was found that these isolates could be separated with 100 % accuracy (Fig. 1b). This basic prediction was to be expected from the obvious differences in genetic content (ΔPV90=1349, ΔPV100=8) and their separation on both core and pan-genome trees. Note that in this situation any human vs bovine signal is masked by more significant serovar/phylogenetic differences. This is shown by adding Dublin human isolates into the analysis; in this case both Typhi and Dublin can still be accurately predicted (100 %), with separation not due to host as the human and bovine Dublin isolates receive the same prediction scores (Fig. 1c).

**Fig. 2.**
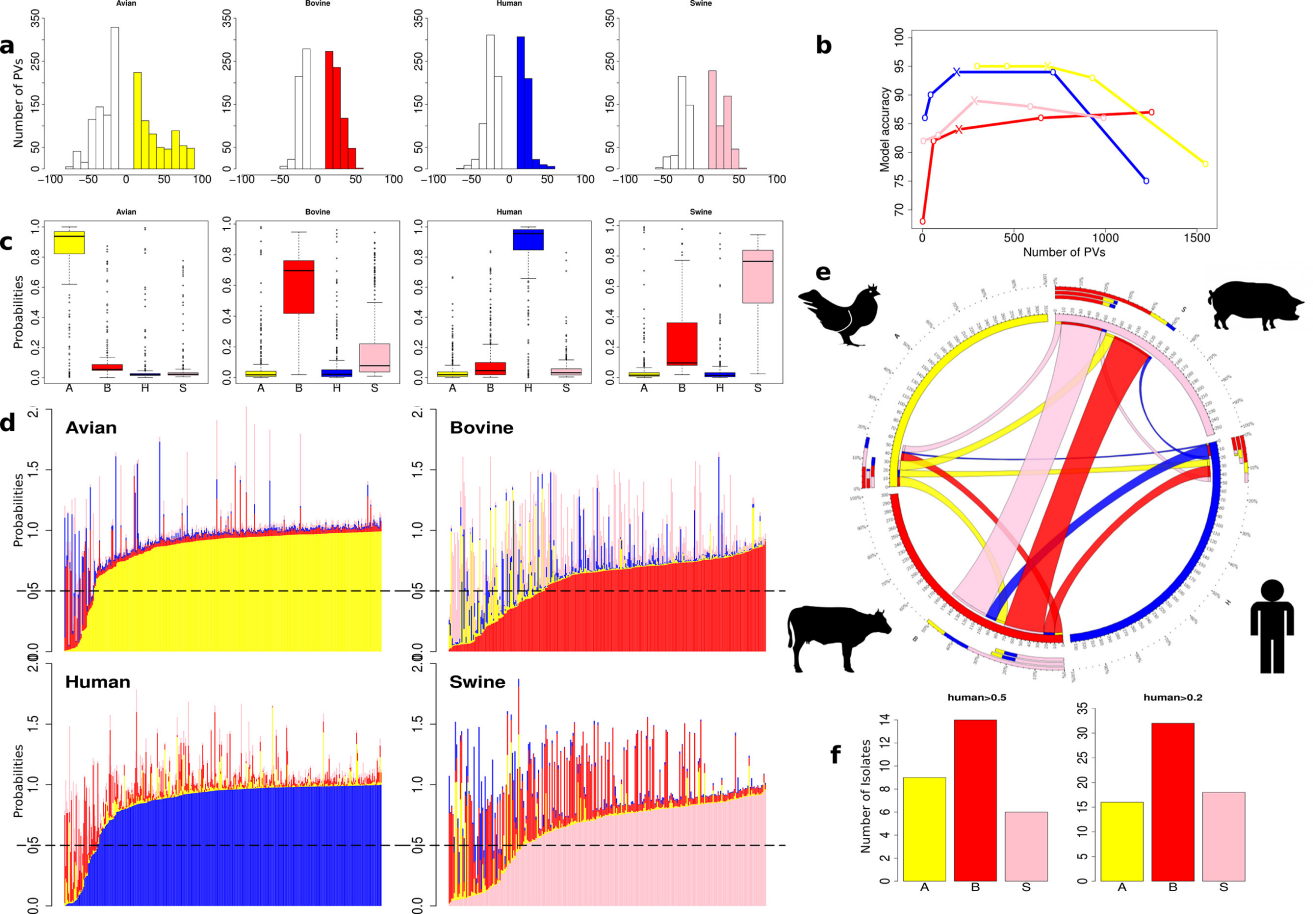
Host prediction by SVM for STm. Colour scheme: STm-avian (yellow); STm-bovine (red); STm-human (blue); STm-swine (pink). (a) The number of and differential PVs on which predictions were based for each host. PVs that differed by less than 10 are not shown (see Methods). The coloured bars are the number of PVs that are present in higher levels in the specified host group, while white bars are the number of PVs more abundant in the ‘all other’ population. (b) Graph showing the relationship between the number of PVs and model accuracy for each host group. Individual points relate to the number of PVs at different ∆PV thresholds from ∆PV>10 to ∆PV>50, plotted from right to left. Crosses define the number of PVs and model accuracy at ∆PV>30, which was applied in the study. (c) Probability assignments of isolate genome content to each host. All STm isolates were tested for their score assignment to each host, expressed as a probability. The sources of the majority of the isolates were predicted correctly, although some hosts have isolates that were more likely to contain genetic information that overlapped with another host. (d) SVM-assigned probabilities for each host plotted for each isolate as a stacked bar. This allows a comparison of the level of host specificity for each isolate. (e) Circos plot depicting the proportion of STm sequence features from each host that can be found in another host. For example, 51 swine isolates with strong porcine prediction scores (>0.5) also had high (>0.5) scores for genetic features from bovine isolates and these are shown as a pink ribbon going from the swine host to bovine host. In total, 52 bovine isolates had a high (>0.5) swine signal and are depicted with a red ribbon going from cattle to swine. The outer ring plots these data as the percentage of isolates assigned other host scores for each specific host. (f) STm isolates scored as human from the different hosts. For each STm isolate the probability of belonging to the human training group was assessed. With a threshold probability of 0.5, there were: nine avian (3 %), 14 bovine (5 %) and six swine (2 %) isolates. When the threshold was set at 0.2, there were 16 avian (5 %), 32 bovine (11 %) and 18 swine (7 %). At this threshold the higher proportion of cattle isolates with human isolate features is significant (Fisher’s exact test: *P*=0.035).

To try to exclude the serovar genetic signal, we tested SVM assignment of isolation host for a single serovar, STm, using the sequences of isolates collected from different sources (human, bovine, avian and swine). From 17 145 total PVs within the STm pan genome, the subtractive difference between the average presence of PVs in a host group versus all others isolates (ΔPV) can be ranked and binned by its discriminatory power (Fig. 2a). The aim was to predict the isolation host with as few PVs as possible while maintaining an acceptable accuracy for the model and its application across all host datasets. A series of test runs were carried out based on different ΔPV values, assessing the quality of prediction and trying to find a ‘one solution fits all’ for the datasets. ΔPV30 was eventually used as a features discriminator for SVM (Fig. 2b). However, it is clear that ΔPV30 is not the best option for all host groups and to further improve predictions for individual studies it would be advisable to choose the ΔPV value according to the dataset.

The main SVM analyses were then carried out by removing a single isolate and training the model with the remainder and then testing that isolate; this process was then repeated until all isolates had been tested. The distribution of discriminatory PVs differed in the four host groups (Fig. 2a): 684 ΔPV30 describe the avian group, 284 PVs for swine, 198 PVs for the bovine group and 182 PVs for the human isolates. Several highly discriminating PVs were identified for the avian group (45 ΔPV80) while for the other host groups there were only a limited number at the ΔPV50 level (bovine=2, human=13, swine=6).

SVM generates a probability, based on comparison of genetic content with the training set, of each test isolate belonging to a specific host group. As such, a logical starting point for our assessment of the methodology for host assignment was to determine how well test isolates could be assigned based on a prediction probability of >0.5 for a specific host. Using this threshold, the majority of the isolates could be classified in relation to their isolation host: 89 % of avian isolates (276 out of 311), 67 % of bovine isolates (202/300), 90 % of human isolates (301/336) and 75 % of swine isolates (192/256) (Fig. 2c, d). The distribution of probabilities was quite distinct for the different host groups. So while the majority of human and avian isolates had high host assignment scores (above 0.8), only a small proportion of isolates achieved such high scores in the bovine and swine groups. The strong SVM assignment for avian and human strains correlated well with their accessory genome clustering (compare Figs 1a and 2c, d). It was also evident that the majority of all isolates achieved a score higher than 0.5 for only one host (94 %), indicating dominant genomic characteristics for this host. However, there were isolates in each host group that scored highly for two or more hosts (total *n*=73), indicating that such isolates, termed ‘generalists’, already contain genetic information that could facilitate existence in at least one other host. Some host groups had low proportions of generalist strains, i.e. the avian and human groups, in which only a minority of isolates were assigned with second host probabilities, >0.5 (human *n*=6, avian *n*=11), while the proportions were much higher among the bovine (*n*=27) and swine (*n*=29) isolates (Fig. 2c, d).

At the left-hand side of each ranked host probability plot (Fig. 2d) are those isolates that have a low probability score for that specific isolation host and would be called as not significantly associated with that host. These isolates often have higher scores for other hosts. This may reflect: (1) the limitation of our strain sets in that we are failing to capture all genetic information relevant to a specific host; (2) incorrect metadata or methodology around collection; and (3) the isolate may be transient and may not persist in that host. It is notable that isolates that are not significantly associated with a particular host (the blanks in the outer ring of Fig. 1a) through the SVM classifier are present in grouped clusters in the pan-genome tree; this includes isolates with a range of host allocations. The implication of this is that particular STm sub-clusters may have a greater potential to switch between specific hosts based on analysis of their genome content.

SVM was used to assign scores to each isolate in relation to its host-relevant genetic content, creating a unique measure of host specificity, and indicates, from this collection of isolates, which animals may be more likely to exchange STm isolates (Fig. 2e). In line with the core and accessory genome trees, the analysis demonstrated a surprising level of host specificity for STm isolates, in particular with avian and human isolates, providing evidence that there may be more human-specific strains circulating beyond our current concerns with ST313. According to this approach, swine and bovine STm isolates can share significant genetic information (Fig. 2e, f).

In each of the three non-human host groups (avian, bovine and swine) there were isolates that achieved an isolation host probability of >0.5 but also reasonable scores for human association. At a 0.5 threshold for human isolate content there was no significant difference between the three hosts, although the highest numbers of such isolates were from the bovine host [avian (*n*=3, 0.9 %), bovine (*n*=9, 3 %), swine (*n*=0, 0 %)]. At a lower threshold (probability assignment >0.2) bovine isolates were significantly more likely to have genetic content associated with human STm isolates when compared with avian and swine isolates [avian (*n*=6, 1.9 %), bovine (*n*=20, 6.6 %), swine (*n*=2, 0.8 %)] (Fig. 2e, f).

We note that as with STm, SVM analysis of bovine and human isolates from within the Dublin serovar can also be predicted with high accuracy (Fig. 1d), as the classifier is again working within the same serovar and so presumably is not confounded by the serovar signal. We then investigated whether it is possible to predict isolation host across serovars, in this case by training on human and bovine STm isolates and testing on human and bovine Dublin isolates, as well as Typhi (Fig. 1e). It was evident that the Dublin isolates could be differentiated by their source even though the training was with STm genome content. Furthermore, *S*. Typhi isolates could be further differentiated in this model based on the STm classifier with significantly higher human association scores (average probability scores for Dublin bovine=0.15, Dublin human=0.27, Typhi=0.48) (Fig. 1e).

###  *Escherichia coli*

**Fig. 3.**
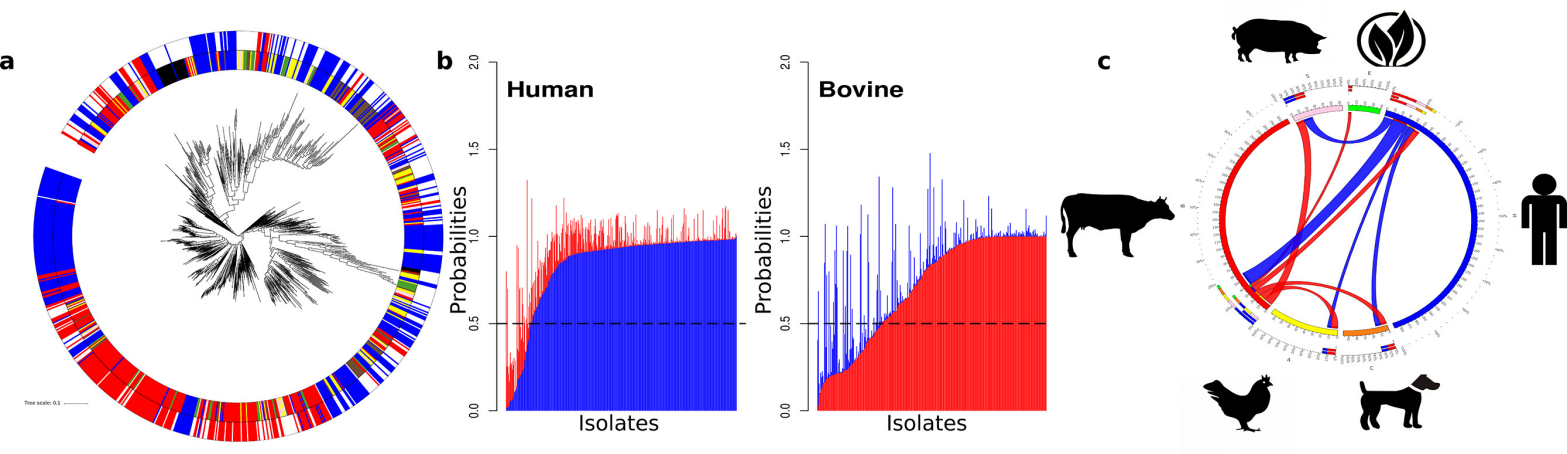
Accessory genome analysis and host prediction by SVM for *E. coli*. Colour scheme: avian (yellow); bovine (red); human (blue); swine (pink). (a) Accessory genome tree based on PVs: some clustering by host for human and bovine isolates was evident. The outer ring indicates the position and isolation host of isolates incorrectly called as human by SVM analysis. (b) SVM host assignment probabilities for human and bovine hosts. The probabilities for each isolate are plotted as stacked bars. (c) The proportions of isolates from each host with human or bovine features.

The *E. coli* dataset was composed of sequences from 943 isolates from six different sources: avian (*n*=87), bovine (*n*=308), canine (*n*=57), environmental (*n*=40), human (*n*=388) and swine (*n*=63). The analysis also included three *Shigella* isolates as these cluster genetically within the *E. coli* species and are considered human-specific. Clustering by relatedness of the accessory genomes is summarized in Table 1. While the number of *E. coli* isolates analysed is almost half that for *S. enterica*, this produced a pan genome that was four times larger than that of *S. enterica*, with more than 90 000 genes. The differences between these two bacterial species were also reflected in the size of their core genome, for which *S. enterica* had almost three-quarters of its genome content shared among the isolates examined while for *E. coli* only one-fifth was conserved across the sequences analysed (Fig. S6). In total, 279 different STs were attributed to *E. coli*, again indicating much greater diversification than for *S. enterica*. A direct comparison of the *E. coli* and *S. enterica* phylogenetic clusters at either accessory (Figs 1a and 3a) or core levels (Figs S5 and S7) indicates a less clear association by host for *E. coli*, although multiple clusters by host were present, especially for human and bovine isolates. Therefore, compared to what was observed for *S. enterica*, there is far more mixing of sub-clusters based on source association for *E. coli*, making prediction of host/habitat attribution more challenging from the accessory genome data presented in this format.

#### SVM prediction of isolation host for *E. coli*

The host/habitat association was predicted based on the SVM approach, in the same manner as for STm. In contrast to the STm analysis, only *E. coli* human and bovine datasets had equivalent isolate numbers so we first tested the impact of reducing dataset sizes on prediction accuracy by working with sub-samples of the larger datasets for both STm and *E. coli*. It was apparent that prediction capacity was substantially reduced when working with fewer than 100 isolate sequences (Figs S1 and S2). Therefore, while predicting the presence of both human and bovine genetic content is valid based on our group sizes, predictions for genetic content pertaining to avian, canine, environmental and swine isolates would require more isolates from these sources to be sequenced and made available. For the larger human and bovine datasets, the prediction capacity was equivalent to that for STm isolates: 72 % (223/308) of bovine *E. coli* isolates and 89 % (346/388) of human *E. coli* isolates were predicted correctly as originating from those hosts based on ΔPV30 (Fig. 3b) using a prediction probability of >0.5.

As with the STm analysis, this indicates a stronger genetic signal for human isolates and greater genetic diversity for bovine isolates. By reducing to ΔPV20 and based on analysis of PV distributions (Fig. S8), the prediction scores for avian, swine and canine *E. coli* isolates show patterns similar to human and bovine isolates when similar size training sets were used (Figs S2, S4, S9 and S10). Therefore, we propose that an equivalent prediction capacity for these sources should be achievable when more isolate sequences are available to train the classifiers. Of note was the pattern for the environmental isolates, half of which showed a strong environmental score while the other half showed a negligible association. This may reflect two different populations of *E. coli* present in the environment, one that is plant/soil associated and the rest more directly related to animals. The ΔPV30 assignments were used to examine human and bovine genetic traits across all the *E. coli* isolates in the study (Fig. 3c). Only a minority of isolates outside of the same host had substantial genetic content associating them with bovine and human hosts (*P*>0.5), which may indicate that only a specific subset may be able to transfer and effectively colonize the two different hosts. Although based on small datasets, it was evident that the inter-relationship between bovine and swine genetic content as seen for STm was not apparent for *E. coli*. Zoonotic potential based on this content can be plotted as for STm. When using a threshold of *P*>0.5, there was a clear and statistically significant hierarchy working towards content in human isolates [environmental (*n*=0, 0 %), avian (*n*=5, 6 %), bovine (*n*=19, 6 %), canine (*n*=7, 12 %), swine (*n*=12, 19 %), Fisher's exact test, *P*=0.002216; Fig. S11)]. The numbers and percentages at a lower threshold of probability, >0.2, were: environmental (*n*=1, 2.5 %), avian (*n*=16, 18 %), bovine (*n*=40, 13 %), canine (*n*=16, 28 %) and swine (*n*=22, 35 %) (Fisher's exact test, *P*=1.023e-05). Independent of the threshold and based on the percentage of isolates (rather than actual number as group sizes varied), porcine isolates had the strongest association with human isolates. Overall, the data indicate that environmental *E. coli* isolates may be less likely to directly infect humans and that bovine, swine and canine isolates are much more likely to be a zoonotic threat than isolates from birds. While this assessment will be refined as more sequences become available, it does demonstrate the utility of the approach.

#### Testing the predictive capacity of SVM with an established bacterial zoonosis

**Fig. 4.**
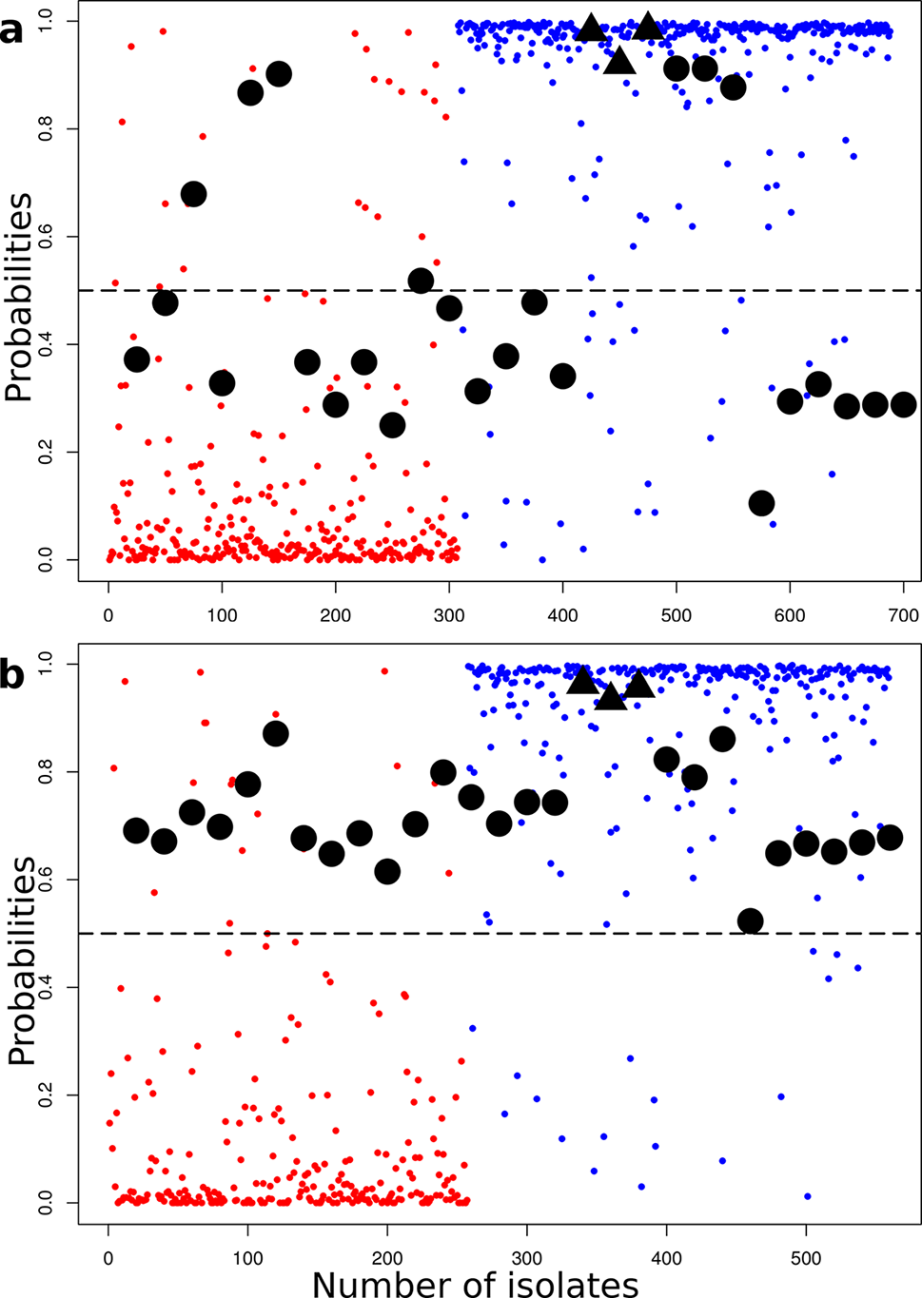
Host assignment of an established bacterial zoonosis: *E. coli* O157. Colour scheme: human (blue) and bovine (red). *E. coli* isolates from both cattle and humans are plotted with their predicted host assignment probability. All these isolates were used as a training dataset to determine host assignment probabilities for O157 isolates (black circles) and three *Shigella* isolates (black triangles). (a) Training set containing *stx*+*E. coli* isolates but not serovar O157, and host assignment probability was then predicted for an O157 test group. (b) Training set with all *stx-*positive isolates removed and the host assigned for the same *E. coli* O157 test group. In both cases the *E. coli* O157 isolates, irrespective of their isolation host, score as containing mixed genetic information in relation to the training set of human and bovine *E. coli* isolates, indicating transmission/zoonotic potential.

As a proof of principle to support the SVM assignments in this study, we determined how machine learning would score sequences from a well-characterized zoonosis. We chose *E. coli* O157 as this clonal group colonizes cattle as an asymptomatic reservoir host and can cause potentially fatal disease in humans as an incidental host. To generate a baseline, all human and bovine isolates from the *E. coli* dataset, but excluding *E. coli* O157 (*n*=688, human=381, bovine=307), were used for SVM training with prediction of host source using ΔPV30 (*n*=139). In this case, training was carried out on 90 % of isolates and testing on 10 % until all isolates had been tested. Overall the source of 92 % of isolates was predicted correctly: 279 of 307 bovine isolates (91 %) and 352 of 381 human isolates (92 %). Most of the isolates were predicted with very high probabilities of originating from human or bovine hosts, with a mean probability of 0.8 (1st quartile of 0.95 and 3rd quartile of 0.98) for human assignments and a mean probability of 0.13 (1st quartile of 0.01 and 3rd quartile of 0.106) for bovine assignments (Fig. 4a). *E. coli* O157 isolates (*n*=25:  14 human, 11 bovine) were then tested in this context along with the three *Shigella* (human isolates) (Fig. 4a). The majority of the probabilities assigned for the O157 isolates were in the mid-range between high human and bovine scores (mean 0.58, 1st quartile 0.44, 3rd quartile 0.73), indicating that the *E. coli* O157 isolates contain ambiguity in their gene content that may allow association with both hosts.

One potential source of bias in the training dataset was the presence of *stx-*positive strains other than O157. Therefore, another analysis was carried out in which *stx*+ isolates were identified and removed from the training dataset (see Methods). The baseline was re-assessed and similar results were obtained with 91.5 % (214 of 234) of bovine isolates and 93 % (266 of 285) human isolates predicted correctly. When probabilities were assigned to the O157 isolates, their distribution changed significantly [mean=0.7203, 1st quartile=0.6520, 3rd quartile=0.7842 (*P*=0.001)] when compared with the previous analysis that included *stx*+ isolates in the training set (Fig. 4b). It is interesting that there were different sets of differential PVs that describe the human and bovine *E. coli* populations depending on the presence (ΔPV30=136) or absence (ΔPV30=248) of *stx*+ isolates. Overall, this result provides strong support for the capacity of the SVM classifier to predict isolates with across-species transmission potential with *E. coli* O157 being assigned probabilities more indicative of human isolates despite cattle being their primary reservoir.

## Discussion

Public repositories of bacterial whole genome sequences, even with very limited metadata, allow new approaches to be tested that address fundamental biological questions such as host specificity and zoonotic potential. In this study we wanted to determine if a machine learning approach, specifically SVM, could assign the isolation host/habitat for both *S. enterica* and *E. coli* isolates based on analysis of differential predicted PVs. Moreover, we wanted to determine if the capacity for inter-species transmission was predictable from the gene content, including estimation of human zoonotic potential. The methods were first applied to *S. enterica* isolates, as serovars such as *S.* Typhi and *S.* Dublin exhibit host specificity and restriction, respectively. As was apparent by both core genome (SNP) and accessory genome analyses, including SVM, *Salmonella* serovars were distinct and easily assigned. By contrast, serovar Typhimurium can be isolated from many different hosts and can cause significant disease in humans with animals often considered the initial source of the infection. Both core and accessory genome clustering provided clear evidence for sub-clusters of STm and several of these were strongly host-associated, in particular for avian and human isolates. Although our analysis is restricted to only a small sample size, it does indicate that host-restricted lineages of STm may extend beyond those receiving attention in relation to their disease severity [22]. The SVM analysis supported these findings with strong host assignment scores for STm isolates. Conversely, only particular sub-clusters contained STm isolates from multiple hosts, and SVM calling of source host in these was much more challenging. However, this does indicate that particular clusters have genetic content that may be more associated with inter-species transmission, indicative of patchy promiscuity within the species.

Certain isolates from each animal host had more genomic content allied with human STm isolates potentially reflecting more of a capacity to infect humans. Overall the bovine STm isolates had the highest predicted ‘human’ scores, even compared with avian isolates. The fact that human STm infections may be more commonly associated with poultry [23] may reflect aspects of the food chain rather than the comparative infection threat of avian STm isolates. In fact, our analysis indicates that the majority of avian STm isolates analysed were quite host-specific and may not pose a public health threat. Support that the SVM classifier was using ‘host-related’ genetic information was provided by training on differential PVs from human and bovine STm isolates and testing on *S.* Dublin from humans and bovine as well as *S.* Typhi from humans. These sets were successfully discriminated by host (Fig. 1e), despite strong phylogenetic signals for the serovar. It is difficult to assess how the phylogeny impacts on the host assignment and in some cases the evolution of particular subtypes may have been driven by host association, in which case phylogenetic and host signatures may overlap. When all isolates from a particular host are combined, the information coming from specific branches/sub-clusters, and therefore the phylogenetic signal, will be diluted and mixed with information from other isolates from other branches. When we then use PVs that describe the ‘avian population’ from these different branches, we decrease the importance of the tree structure. For example, we can predict avian strains from different regions of the tree despite there being a dominant avian isolate cluster. A primary driver for this publication is to demonstrate the potential for machine learning alongside phylogeny approaches, and the value and relationships between these will become apparent as sequences of more isolates from different sources become available.

*E. coli*, in comparison to *S.* Typhimurium, had more limited host-specific sub-clusters based on core and accessory genome analyses, although it was still possible to correctly call the host of origin for the more populated datasets of bovine and human isolates using the SVM classifier. We included isolates from other hosts/habitats to provide more discriminatory power in the ‘one host vs all approach’ but again prediction accuracy for *E. coli* from different sources will increase as more of these host/habitat-related sequences are made available (Fig. S2a, b). Even so, it was evident that environmental *E. coli* isolates had very little overlap with human isolates and that human infection may therefore be more likely from animal-adapted *E. coli* isolates. With the SVM approach, bovine, swine and canine isolates all had subsets that shared significant genetic content with human isolates. The analysis of *E. coli* O157 isolates provided validation that the SVM classifier, trained on bovine and human *E. coli* isolates, could identify isolates with increased zoonotic potential, as isolates of this established zoonotic clone produced intermediate scores reflecting mixed genetic assignment between other human and bovine isolates.

Both STm and *E. coli* isolates exhibited marked host restriction when genetic content was evaluated using a combination of phylogenetic and machine learning methods. We consider this is counter to a perception that these bacteria are ‘generalists’ capable of switching between hosts. Instead, our analyses indicate that only specific subsets of strains have ‘mixed’ genetic content, which we suggest indicates the capacity to transfer and succeed in different hosts, although this now needs to be tested using experimental approaches. We consider that machine learning has tremendous potential to interrogate complex seqLineColumnRule IDProbe MessageNode TextNode XpathParent Node Textfatal/var/www/html/_default/resources/microbio/__package/144333/144333.xmlf002block-formatting check: Entire content of title should not be formatted (Tagging Guidelines)Salmonella entericauence datasets and identify genes/sequences associated with host specificity. This will have value for source attribution in both a public health context and, for example, in ascribing the source of water pollution events if sequences of the bacteria are obtained.

## Data bibliography

1. Figshare, https://figshare.com/s/7a3ededa8cedd95b9fb7 (2017).

## Supplementary Data

1020 Supplementary File 1
