# Erratum: Patchy promiscuity: machine learning applied to predict the host specificity of *Salmonella enterica* and *Escherichia coli*

**DOI:** 10.1099/mgen.0.000193

**Published:** 2018-06-06

**Authors:** Nadejda Lupolova, Tim J. Dallman, Nicola J. Holden, David L. Gally

**Affiliations:** ^1^​ University of Edinburgh, Edinburgh, UK; ^2^​ Public Health England, England, UK; ^3^​ James Hutton Institute, Dundee, UK; ^4^​ Division of Immunity and Infection, The Roslin Institute, University of Edinburgh, Easter Bush, Edinburgh EH25 9RG, UK

An error occurred during the publishing process of this article.

There was text inserted in the final paragraph of the Discussion, in the following sentence:

‘We consider that machine learning has tremendous potential to interrogate complex seqLineColumnRule IDProbe MessageNode TextNode XpathParent Node Textfatal/var/www/html/_default/resources/microbio/__package/144333/144333.xmlf002block-formatting check: Entire content of title should not be formatted (Tagging Guidelines)Salmonella entericauence datasets and identify genes/sequences associated with host specificity.’

The sentence should read as follows:

‘We consider that machine learning has tremendous potential to interrogate complex sequence datasets and identify genes/sequences associated with host specificity.’

The Microbiology Society apologizes for any inconvenience caused.

