# Supplementary material for: Patchy promiscuity: machine learning applied to predict the host specificity of Salmonella enterica and Escherichia coli
Source: Microb Genom. 2017 Oct 3;3(10):e000135. doi: 10.1099/mgen.0.000135 (PMC5695212; doi:10.1099/mgen.0.000135)
Supplement: Supplementary File 1 [file mgen-3-135-s001.pdf]

Additional file 1

Patchy promiscuity: machine learning applied to predict the host specificity of *Salmonella enterica* and *Escherichia coli*

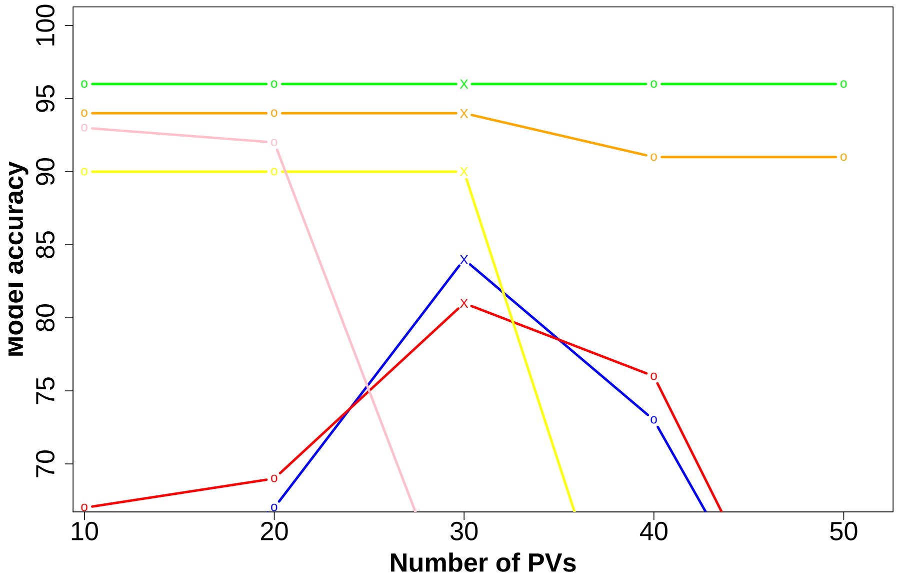

Figure S1: **Model accuracy vs. number of PVs for *E. coli*.** Each point from left to right indicates ΔPV50, ΔPV40, ΔPV30 (shown as crosses, these were chosen for the final model), ΔPV20, ΔPV10. The aim was to find a value that could be used for all the training models within the *E. coli* set, but it is clear that a "one fits all" is not the best strategy for this particular analysis. It is evident that the same threshold as applied to STm (ΔPV30) challenging to use for all *E. coli* sub datasets as in some of them (swine and avian) were too few PVs available. Similar to the *Salmonella* dataset, this analysis indicates that increasing the number of ΔPVs does not always lead to an increase in accuracy of the model.

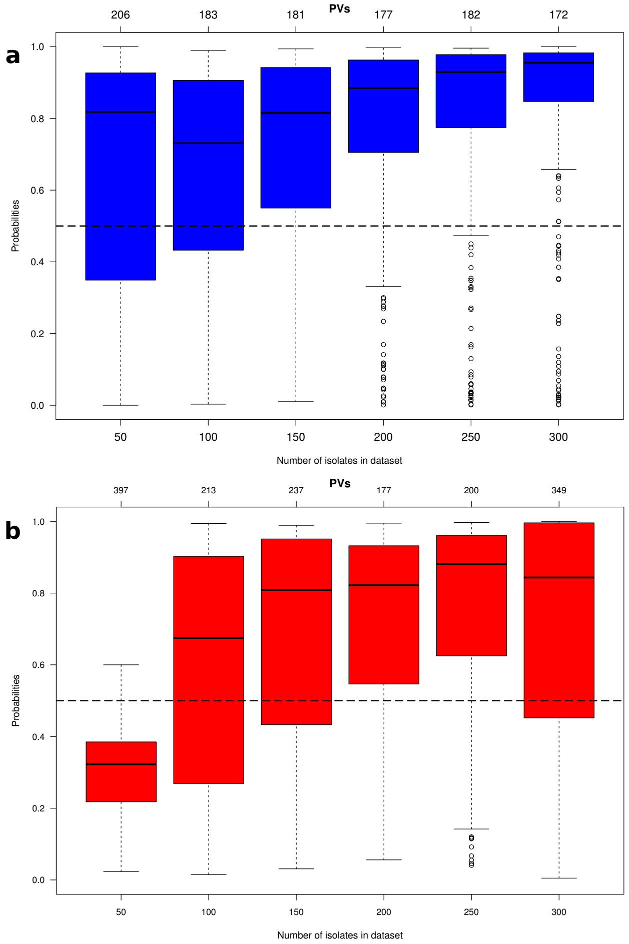

Figure S2: **Influence of dataset size on the number of PVs and prediction accuracy.** **(a)** Boxes represent predictions for gradually increasing number of *S*. Typhimurium human isolates, while the number of bovine isolates is kept constant. **(b)** The same as above with an increasing number of bovine *E. coli* bovine isolates and a constant number of human isolates. Increasing the number of isolates in the dataset mostly improves predictions.

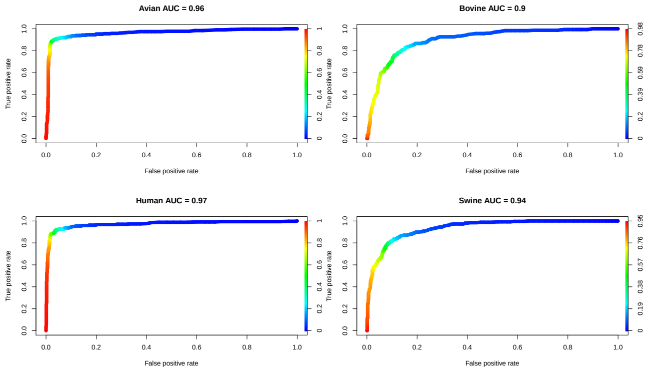

Figure S3: **Performance of SVM models for *S.* Typhimurium isolates.** Area under the curve illustrating performance of four classifiers for each host model for *S.* Typhimurium dataset.

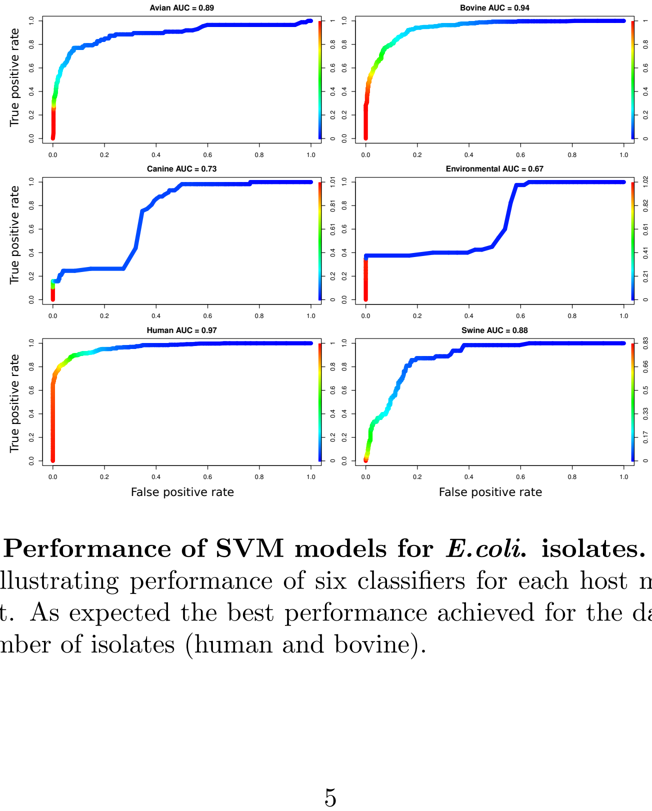

Figure S4: **Performance of SVM models for *E.coli.* isolates.** Area under the curve illustrating performance of six classifiers for each host model for *E. coli* dataset. As expected the best performance achieved for the datasets with highest number of isolates (human and bovine).

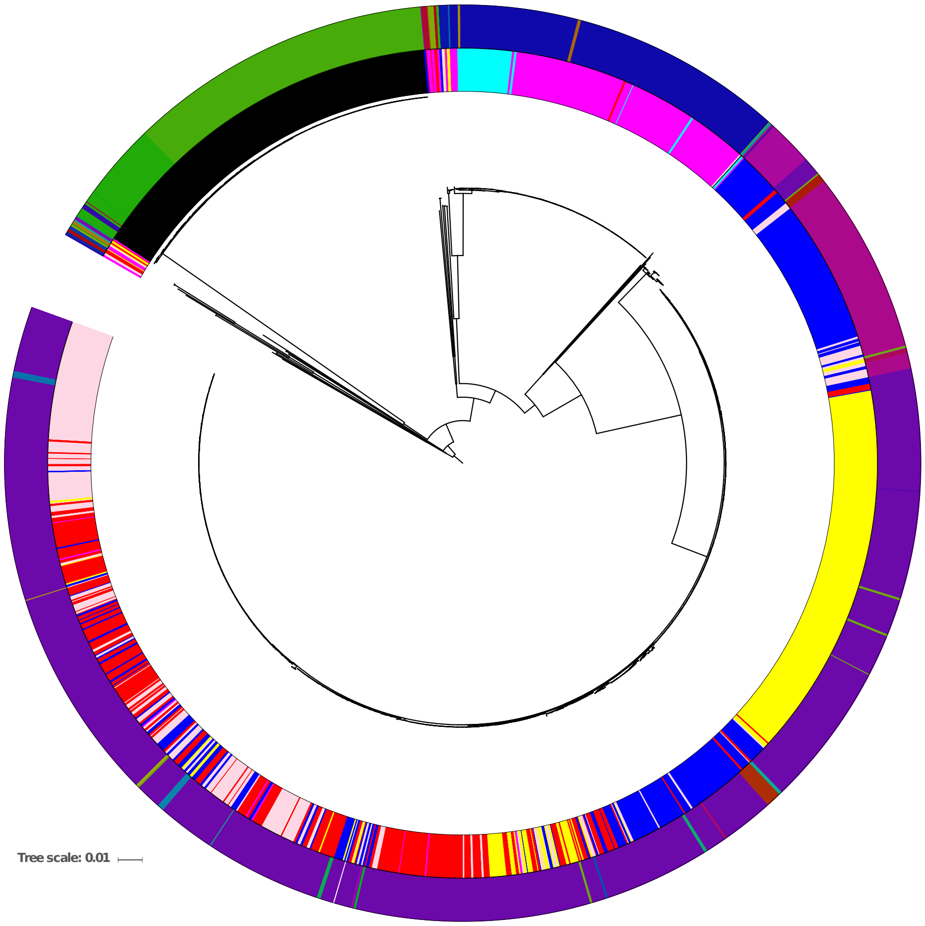

Figure S5: ***S. enterica* core genes tree**. Maximum likelihood core genes tree with host and serovar information shown in the inner circle (blue-human STm; yellow-avian STm; red-bovine STm; pink-porcine Stm; black-S.Typhi; dark pink-bovine *S.* Dublin; cyan-human *S.* Dublin) and MLST Sequence Type information in the outer circle.

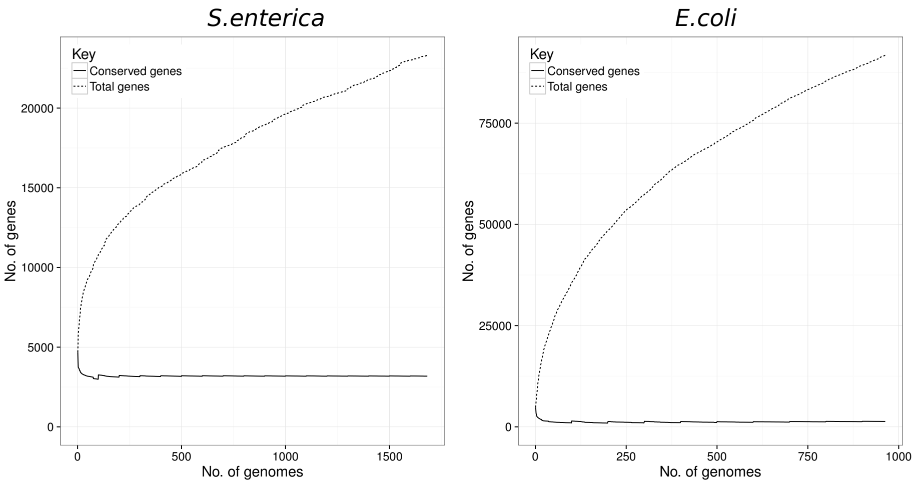

Figure S6: **Pan genome sizes of *S. enterica* and *E. coli*.** The figure illustrate the differences in pan-genome structures for *S. enterica* and *E. coli*. Even though almost only half as many isolates were analysed for *E. coli* (n = 943) compared to *S. enterica* including Typhi and Dublin (n = 1682), *E. coli* had a pan-genome that was 4 times the size of pan- genome of *S. enterica*

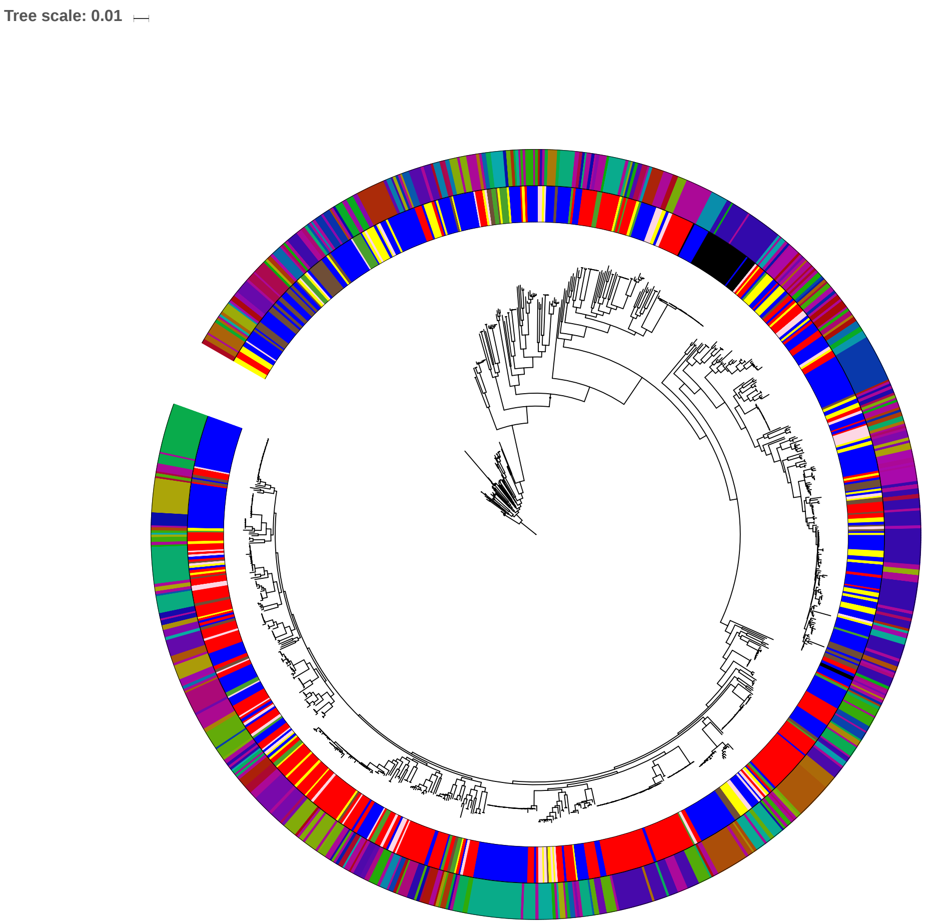

Figure S7: ***E. coli* core genes tree** with host information shown in the inner circle (blue-human; yellow-avian; red-bovine; pink-porcine; green-environmental; brown-canine) and Multi Locus Sequence Type-MLST information shown in the outer circle.

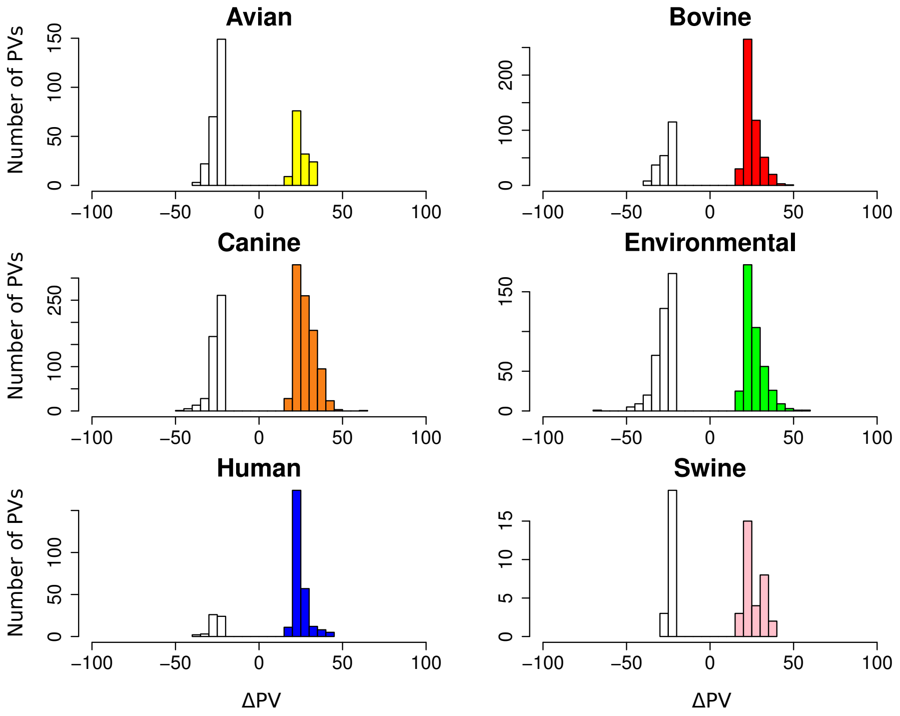

Figure S8: **Distribution of descriptive PVs for *E. coli***. The number of PVs is shown on the Y axis and the ΔPV range on the X axis with positive values indicating increased presence of the PV in the defined host group and negative values meaning increased presence of the PV in the remainder.

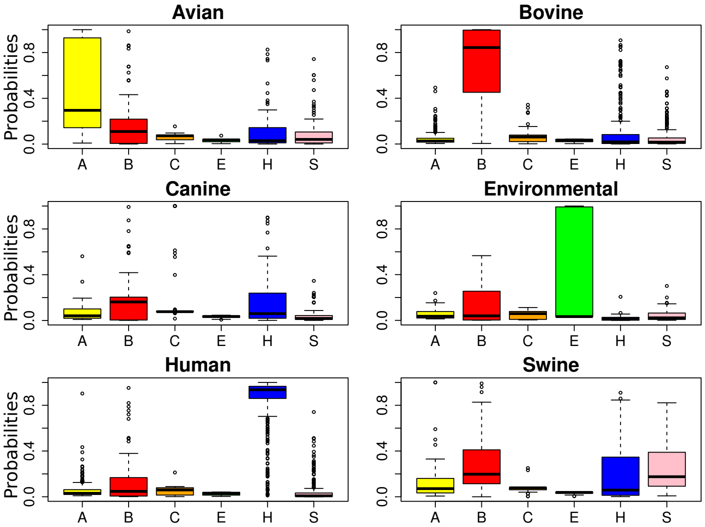

Figure S9: ***E. coli* boxplot predictions.** Distribution of probabilities of *E.coli* isolates plotted as a boxplot for each host. Color scheme: yellow - avian, red - bovine, orange - canine, green - environmental, blue - human, pink - swine.

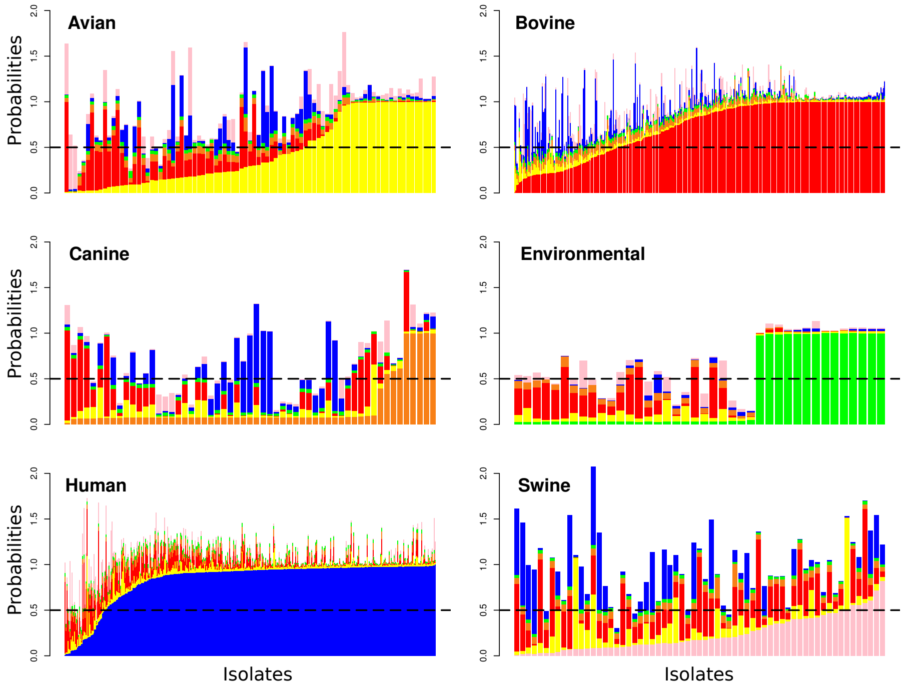

Figure S10: ***E. coli* prediction of host assignment plotted as stacked bar-plots.** As discussed in the main text, the lack of specific assignment for all hosts/environments other than bovine & human may be due to lack of isolate data and so care needs to be taken in interpreting these graphs. It is evident that the environmental group does have a very different structure and indicates a subset of *E.coli* with a strong environment-specific attribution.

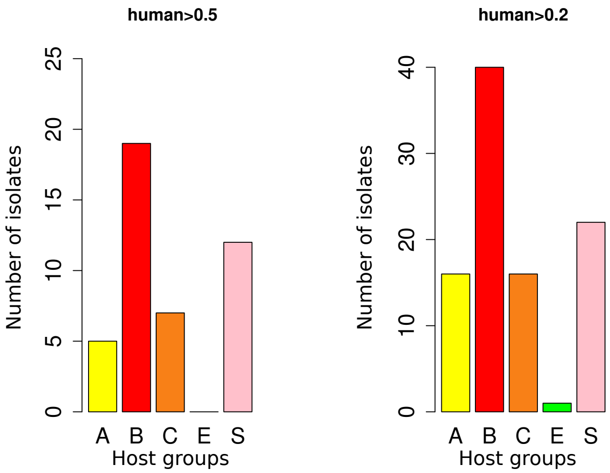

Figure S11: ***E. coli* isolates scored human**. X-axis host groups, Y-axis number of isolates. There was a clear and statistically significant hierarchy working towards content in human isolates (environmental(n=0, 0%), avian(n=5, 6%), bovine(n=19, 6%), canine(n=7, 12%), swine(n=12, 19%), Fisher's Exact Test, p-value = 0.002216. The relative numbers at the p> 0.2 threshold were: environmental(n=1, 2.5%), avian(n=16, 18%), bovine(n=40, 13%), canine(n=16, 28%), swine(n=22, 35%), Fisher's Exact Test: p-value = 1.023e-05.

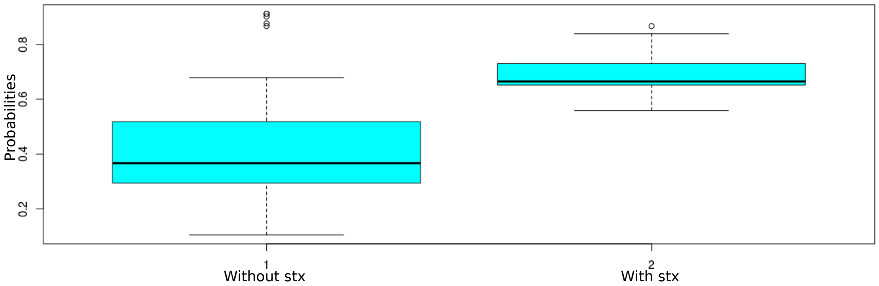

Figure S12: ***E. coli* O157 isolates predictions**. The figure illustrates how 'human isolate' predictions changed when 24 *E. coli* O157 isolates were tested on either all *E. coli* human and bovine isolates (with stx) as the training sets or with stx+ containing isolates removed from these two training sets (without stx).
